# Measuring contraceptive use in a displacement-affected population using the Multiple Indicator Cluster Survey: The case of Iraq

**DOI:** 10.1016/j.jmh.2022.100114

**Published:** 2022-05-12

**Authors:** Rosanna Le Voir

**Affiliations:** Department of Methodology, London School of Economics and Political Science, Houghton Street, London WC2A 2AE, United Kingdom

**Keywords:** Displacement, Contraception, Internally displaced persons, Sexual and reproductive health, Household surveys, Iraq

## Abstract

Access to safe, effective, affordable, and acceptable contraceptive methods of choice is a basic right for displaced people. Yet displaced people are typically invisible in national sample surveys on population health, and quantitative evidence on their reproductive health outcomes is limited. This study focuses on the case of Iraq, a country with widespread displacement and where contraceptive use is a government policy priority. Using displacement screening questions in the Iraq 2018 Multiple Indicator Cluster Survey questionnaires, I construct two displacement-related indicators based on reason for last move and previous household residence. Descriptive statistics and binary logistic regression are used to test associations between modern contraceptive use and displacement, demographic, and socioeconomic factors. Controlling for the variables in the models, factors significantly associated with modern contraceptive use among married women aged 15–49 in Iraq are Federal Iraq region (reference Kurdish Region of Iraq, OR 1.78), upper secondary and primary education (reference pre-primary or no education, OR 1.50 and 1.20, respectively), parity, age, and exposure to television. The association between displacement (reason for last move) and modern contraceptive use significantly depends on a woman's level of education and whether they live in an urban or rural area. Women who previously lived in a camp are almost half as likely to use modern contraception compared to other previous residence types. This paper highlights the methodological potential and substantive value of using national household surveys to analyse reproductive health outcomes through a displacement lens. It also critically examines the limitations of these data and measures, drawing on total survey error and feminist theory.

## Highlights

• Offers substantive and methodological contributions to the literature on IDP health.
• Uses Multiple Indicator Cluster Survey data with displacement screening questions.
• Tests associations between modern contraceptive use and displacement in Iraq.
• Total survey error and feminist approaches highlight the limits of national household surveys.

## 1 Introduction

### 1.1 Situating displacement in “development” data and targets

Sustainable Development Goal (SDG) targets 3.7 and 5.6 call for universal access to sexual and reproductive health services and rights (UN Economic and Social Council, 2015). Some of the slowest progress towards health-related SDG goals are in countries affected by humanitarian crises (Sachs et al., 2021). In these contexts, forcibly displaced people, particularly internally displaced persons (IDPs), are some of the most vulnerable (Zeender, 2018). Yet displacement is almost invisible in global development frameworks such as the SDGs.

This statistical invisibility may be because displacement is difficult to conceptualise and measure (Joint Data Center on Forced Displacement, 2020). Collection of high quality data on IDPs is limited by conceptual, operational, and political challenges (Baal and Ronkainen, 2017). These range from variations in definitions of IDPs - a current priority of the Expert Group on Refugee, IDP and Statelessness Statistics (EGRISS) - to high population mobility and insecurity (Expert Group on Refugee and Internally Displaced Persons Statistics (EGRIS), 2020). IDPs are often excluded from national data systems and planning. Evidence on displacement-affected populations is mainly produced by humanitarian sources for operational purposes, such as the United Nation's Humanitarian Needs Overview (United Nations, 2021). There is limited comparative analysis of IDPs within the wider population.

The main sources of data on reproductive health in low- and middle-income countries are internationally comparable and nationally representative cross-sectional household surveys, such as the Multiple Indicator Cluster Survey (MICS) and Demographic and Health Survey (DHS). The metrics measured in these surveys serve as a common numerical language among experts, advocates and bureaucrats (Wendland, 2016, Merry, 2016). However, DHS and MICS are rarely conducted in humanitarian contexts, and questions on displacement status are not typically included (UNHCR, 2020). Survey reports include only limited breakdown by sub-population with little recognition of inequalities within countries (Galati, 2015), including IDPs. As such, displacement has been neglected as an explanatory factor in analyses of reproductive health outcomes, and there is a gap in understanding reproductive health outcomes in humanitarian contexts (Blanchet et al., 2017).

### 1.2 Reproductive health in displacement

Reproductive health was defined at the foundational International Conference on Population and Development in 1994 as “a state of complete physical, mental and social well-being and not merely the absence of disease or infirmity, in all matters related to the reproductive system and to its functions and processes” (United Nations, 2014). Reproductive health is a human right, central to gender equality, wider positive health outcomes, and sustainable development (Starrs et al., 2018). Access to safe, effective, affordable and acceptable contraceptive methods of choice, is a key component of basic services in humanitarian contexts, including for IDPs (UNFPA, 2020).

The available peer-reviewed qualitative literature highlights inadequacies in reproductive health services among displacement-affected populations and humanitarian contexts. For example, a recent Lancet series on women's and children's health in conflict settings found that across 10 case studies, most reproductive health services were reportedly not delivered (Singh et al., 2021). This contrasts with other services that were prioritised, including nutrition, maternal and child health.

Displacement-affected populations are exposed to specific risks such as weak health service provision and systems access (Ager, 2014) that may influence reproductive health outcomes (Kismödi and Pitchforth, 2022). The risks and vulnerabilities vary depending on their socioeconomic situation and demographics. A scoping review in this issue highlighted that IDPs generally experience worse health outcomes compared to other populations affected by conflict (Cantor et al., 2021). Case study evidence from Ethiopia identified age- and gender-specific vulnerabilities among IDPs (Jones et al., 2021). Furthermore, missing or inadequate civil registration documents, a common issue for IDPs, can prevent access to public services (Saieh, 2019). This may be particularly problematic for poorer IDPs, whereas wealthier households may be able to pay for private services instead. Access to services may also vary across geographic areas, including urban and rural, and for populations living in displacement camps compared to those in the community.

Existing reviews have highlighted evidence gaps on contraceptive patterns at both utilisation and outcome level in humanitarian contexts (Singh et al., 2018). The World Health Organisation recently identified contraceptive services in low- and middle-income countries in general as a global research priority (Kobeissi et al., 2021). A limited number of studies document reproductive health needs and outcomes among displaced people in the Middle East, primarily focusing on refugees (Balinska et al., 2019; Tanabe et al., 2017; Reese Masterson et al., 2014; Amiri et al., 2020; Krause et al., 2015). I was unable to find any quantitative studies that used national survey data to estimate contraceptive use among displaced people in the region.

### 1.3 Relevance of the Iraq case

Iraq is an instructive country to study contraceptive use among displacement-affected populations. Firstly, in case study research typology, Iraq is an “extreme” case (Flyvbjerg, 2006) of displacement. Between 2010 and 2019, Iraq accounted for one in five of the world's total displaced people due to conflict and disasters (Anzellini et al., 2021). Iraq's displacement crisis is characterised by both refugee and IDP movements. Displacement is not a new phenomenon in Iraq; there have been numerous spikes in recent decades. One key wave was linked to events during Saddam Hussein's era, particularly the 1980–88 Iran-Iraq war, Al-Anfal campaign, and the 1991 first Gulf War, with an estimated one million IDPs by 2003 (International Organization for Migration, 2018). Further displacements from 2003 were associated with the removal of Saddam Hussein's regime and the ensuing sectarian violence, as well as the bombing of the holy Samarra shrines in 2006 (Lafta and Al-Nuaimi, 2019). The situation stabilised between 2008 and 2012, with the number of IDPs reducing from an estimated 2.7 million people at the end of 2008, to around 1.3 million by September 2012 (International Organization for Migration, 2018). The conflict with ISIS between 2014-17 triggered a displacement crisis on an even greater scale. More than six million Iraqis were internally displaced since 2014, comprising around 15% of the population (IOM Iraq, 2021). Some of the most affected governorates were Baghdad, Salah al-Din, Diyala, Ninewa, and Anbar. The International Organization for Migration (IOM) estimates that around 1.2 million people remain internally displaced as of 2021 (International Organization for Migration, 2021). This is in addition to around 4.9 million people who were displaced and have now returned to their area of origin, so called “returnees”.

Contraceptive use has emerged as a national policy priority in Iraq. The country is a demographic anomaly in the region, with a high fertility rate comparable to Yemen and Gaza, and a large, young and rapidly growing population (Hamilton, 2020). This can be traced to historical pronatalist policies during Saddam Hussein's regime, particularly through the Iran-Iraq war, including childbirth cash bonuses and limits to contraceptive services (Cetorelli, 2014). Years of conflict, sanctions, under-investment, and loss of health workers have eroded the health system (al Hilfi et al., 2013). The Iraqi government launched a new Family Planning and Birth Spacing Strategy 2021–25 in October 2020 (Iraq Ministry of Health, 2020). While contraception is available in Iraq, including free services at government health centres, there is limited evidence on its uptake (al Ameen and al Deen, 2016). Knowing the patterns and predictors of contraceptive use among the population, including IDPs, will be critical to understanding the impact of this strategy.

Finally, and unusually for a humanitarian context, national household survey data on reproductive health outcomes, including contraceptive use, are available for Iraq. MICS is a nationally representative cross-sectional sample survey conducted by the Iraq Central Statistical Organization and the Kurdistan Region Statistical Office, in coordination with the Ministry of Health and UNICEF (Iraq Central Statistical Organisation, Kurdistan Regional Statistics Office, 2019). A set of displacement screening questions were included in the 2018 Iraq MICS.

### 1.4 Focus of the study

This study uses the case of Iraq to illustrate the methodological potential and substantive value of using national household surveys to analyse reproductive health outcomes through a displacement lens. Specifically, it: (1) explores feasibility of constructing “displacement” measures based on the screening questions in the 2018 Iraq MICS questionnaires; (2) tests quantitative associations between displacement and modern contraceptive use; and (3) discusses the limitations of existing surveys for analyses of reproductive health outcomes among displacement-affected populations.

## 2 Material and methods

### 2.1 Data sources

This study uses secondary quantitative survey data from the 2018 Iraq MICS (Iraq Central Statistical Organisation, Kurdistan Regional Statistics Office, 2019). The timing of data collection is highly relevant, following the elevated levels of displacement between 2014 and 2017 in Iraq (International Organization for Migration, 2018). Surveys such as MICS, with a robust design and large sample sizes, are considered high quality evidence in global health (Parkhurst, 2017).

MICS uses a standard complex survey design. For the Iraq 2018 MICS, the *target population* covers all 18 governorates of Iraq, including Federal Iraq and the Kurdish Region of Iraq (KRI). The *survey population* - constrained by resource and practical considerations - excludes some hard-to-reach groups, including IDPs living in formal camps. IDPs living outside of camps are included. The *frame population* is based on a 2009 update of the sampling frame developed for the last census in 1998. The survey sample is selected using a multistage, stratified cluster sampling approach.

The Iraq 2018 MICS administered five face-to-face questionnaires using interviewers. This study uses data from two questionnaires: the main household questionnaire and individual questionnaire for all women aged 15–49 in each household. Of the 20,520 households sampled in the 2018 Iraq MICS, 31,060 women aged 15–49 were eligible to be interviewed. Of these, 30,660 were interviewed, representing a response rate of 98.7%. This is slightly lower than the main household questionnaire response rate of 99.5%, but comparable with household surveys previously conducted in Iraq (Iraq Ministry of Planning, 2012). Questions on contraceptive use were only administered to women who reported that they were currently married or living with a man, comprising a sample size of 19,597.

The MICS Technical Committee advises on ethical issues and approved the survey protocol. MICS data are publicly available pending free registration.

### 2.2 Measures

#### 2.2.1 Response variable – modern contraceptive use

The response variable was modern contraceptive use, measured by modern contraceptive prevalence rate (mCPR). CPR is defined as the percentage of women of reproductive age (15−49 years), married or in union, who are currently using, or whose sexual partner is using, at least one method of contraception (World Health Organization, 2018). I focused on modern methods of contraception, including condom, pill, injectable, implant, intrauterine device, and sterilisation (United Nations, 2019), rather than any method, since these methods are more dependent on health systems access that may be disrupted by displacement. This attention to modern methods aligns with the emphasis of the SDGs and the Iraq National Birth Spacing and Family Planning Strategy. The limitations of this narrow binary indicator have been highlighted by feminist scholars (Senderowicz, 2020), but it remains one of the most common measures of reproductive health globally.

The indicator was operationalised in MICS using two questions in the individual women's questionnaire (Table 1). I constructed the response variable, using modern contraception, with values any modern method coded as 1 (response options A-K), and otherwise coded as 0 (response options L-X or missing).

$$Y=\{\;\begin{array}{c} 1\;(\text{any}\;\text{modern}\;\text{method})\\ 0\;\left(\text{otherwise}\right) \end{array}$$

**Table 1:** Questions in the 2018 Iraq MICS individual women's questionnaire on contraceptive use.

Variable name	Question	Response options
CP2	Couples use various ways or methods to delay or avoid getting pregnant. Are you currently doing something or using any method to delay or avoid getting pregnant?	Yes: 1 No: 2
CP4	What are you doing to delay or avoid a pregnancy?	Female sterilization: A Male sterilization: B IUD: C Injectables: D Implants: E Pill: F Male condom: G Female condom: H Diaphragm: I Foam/jelly: J Lactational amenorrhoea method: K Periodic abstinence/rhythm: L Withdrawal: M Other: X

#### 2.2.2 Explanatory variables – displacement

I reviewed the 2018 MICS questionnaires to identify all options for constructing variables on displacement. Based on the available questions, I generated two binary explanatory variables from the household questionnaire, relating to (1) reason for last move and (2) previous household residence (see Table 2). The sub-population for these variables is where the respondent (head of the household) has not lived in the same place since birth.

**Table 2:** Categorisation of responses to questions HC2E and HC2B in the household questionnaire.

Question	Category	Response option
HC2E. What was the main reason for moving?	Displaced	Conflict or violence Tribal land disputes Government evictions Natural disasters Return home
	Other reason for moving	Economic reasons Education Family reunification Other
HC2B. Just before moving here, did (name of the head of the household from HL2) live in a city, in a town, in a rural area or in a camp?	Camp	Camp
	Other residence	City Town Rural area

The variable about reason for last move was labelled *displaced* and based on question HC2E. Responses were coded 1 (displaced) when the head of household reported the main reason for their last move of residence as conflict or violence, tribal land disputes, government evictions, return home, or natural disasters. Other response options were categorised as 2 (other reason for moving), including economic reasons, education, family reunification, or other. This categorisation aligned the MICS screening questions with the UN Guiding Principles definition of internal displacement, as the movement of persons (1) within national borders, (2) in anticipation of or in response to specific risks, particularly situations of armed conflict, human rights violations or natural or human-made disasters (United Nations, 1998). The challenges of operationalising this definition for statistical purposes are well documented (Baal et al., 2018). The questionnaire does not ask about place of birth, so a key assumption is that displaced people are internally displaced. I used empirical comparisons with other measures of displacement at the national level for sensitivity analysis. For example, IOM estimates 15% of the total population were displaced between 2014 and 2017; based on my categorisation of the 2018 MICS response options, 16% of the population of married women were estimated to be displaced.

The second explanatory variable relating to previous household residence (question HC2B) was labelled *camp*. Response options “city”, “town” and “rural area” were coded as 2 (other residence), and “camp” was coded as 1.

#### 2.2.3 Other explanatory variables – demographic and socioeconomic characteristics

Existing literature from low- and middle-income countries shows that modern contraceptive use varies by socioeconomic and demographic factors at the household and individual level (Wulifan et al., 2016). I reviewed the 2018 Iraq MICS questionnaires to identify all relevant predictors of modern contraceptive use, drawing on both the household and individual women's questionnaire. Table 3 outlines the full set of explanatory variables.

**Table 3:** Displacement, demographic, and socioeconomic explanatory variables.

Domain	Explanatory variable	Operational definition
Displacement	Reason for last move	Self-reported main reason for last move, categorised as displaced (1) or other reason for move (2)
	Previous type of residence	Self-reported previous residence, categorised as camp (1) or city/town/rural area (2)
Demographic	Age of woman	Self-reported age of woman in years at time of survey (range 15-49), categorised into five year age groups
	Parity	Self-reported number of children ever born, categorised into groups: 0, 1, 2, 3, 4, 5+
	Region	Region of Iraq, categorised as Federal Iraq or KRI
	Governorate	18 subnational administrative areas
	Area	Rural/urban
Socioeconomic	Highest education level	Self-reported highest level of education completed by woman, categorised by pre-primary or none, primary, lower secondary, upper secondary+
	Wealth quintile	Poorest, second, middle, fourth, richest
	Media exposure	Self-reported frequency watching television by woman, categorised by not at all, <1 per week, ≥1 per week, almost every day

### 2.3 Hypotheses

I expected modern contraceptive use to show a negative association with displacement. This is because displacement and conflict can disrupt access to health commodities and weaken the local health system (Busza and Lush, 1999; Miliband and Teklu Tessema, 2018). By contrast, health system access among those moving for other reasons (such as economic or education motivations) may not be affected in the same way (Abubakar et al., 2018).

I expected the association between modern contraceptive use and displacement to vary across demographic and socioeconomic characteristics. I anticipated a statistically significant relationship between modern contraceptive use and factors such as woman's age and parity, due to their intention to limit or space births at different stages of their reproductive life (Bongaarts and Potter, 1983). Recognising the socioeconomic heterogeneity of IDPs, I expected the association between displacement and modern contraceptive use to depend on factors such as area, wealth, and education. This is because health-related risks and choices may vary across IDPs and be experienced differently by poor or wealthy households, level of education, or people living in different areas.

### 2.4 Data analysis

Statistical analyses were conducted using Stata 16 software (StataCorp, 2019). I calculated frequencies and proportions to describe the sub-population of interest, married women aged 15–49 years, reporting point estimates and 95% confidence intervals. I checked for missing data and small numbers of observations that could make the model unstable and violate the assumptions of maximum likelihood estimation.

Descriptive statistics were used to analyse modern contraceptive use by sociodemographic characteristics. Crosstabs between the response variable and categorical explanatory variables informed my subsequent analyses. For example, it would have been interesting to explore associations between modern contraceptive use and different reasons for displacement, but the number of married women aged 15–49 in these categories was too small (e.g. natural disasters, *n* = 11).

I conducted sensitivity analyses comparing CPR (modern) and CPR (any method) as the response variable. I found that CPR (any method) did not vary between the displaced and wider population. Based on reason for last move, CPR (any method) was 52% among both displaced households and those who moved for other reasons, and 53% for households who never moved. For those who previously lived in a camp, CPR (any method) was 51%, compared to 52% among people who previously lived in other residences. This supported the focus on modern contraceptive use in the regression models.

I used binary logistic regression to model variation in the response variable (*modern contraceptive use)* with displacement, demographic, and socioeconomic explanatory variables. This study did not aim to draw causal inferences, so I use terminology such as *predictors* or *factors associated with* modern contraceptive use, rather than *determinants*. I applied a single critical value of 5%, such that where *p* < 0.05, the association was counted as significant. Ultimately three models were used:

1 Bivariate model to test for an association between the two displacement-related variables and modern contraceptive use:
  $$\text{Logit}\;(\text{modern})\;=\;\alpha \;+\;\beta_{1}\text{displaced}$$
  $$\text{Logit}\;(\text{modern})\;=\;\alpha \;+\;\beta_{2}\text{camp}$$
2 Multivariate model with all relevant displacement, demographic, and socioeconomic explanatory variables to test for associations with modern contraceptive use:
  $$\text{Logit}\;(\text{modern})\;=\;\alpha \;+\;\beta_{1}\text{displaced}\;+\;\beta_{2}\text{camp}\;+\;\beta_{3}\text{age}\;+\;\beta_{4}\text{region}\;+\;\beta_{5}\text{governorate}\;+\;\beta_{6}\text{area}\;+\;\beta_{7}\text{education}\;+\;\beta_{8}\text{wealth}\;+\;\beta_{9}\text{parity}\;+\;\beta_{10}\text{media}$$
3 Multivariate model including all sociodemographic explanatory variables in (2), as well as interactions. For example, an interaction between displacement (reason for moving) and education level:
  $$\text{Logit}\;\left(\text{modern}\right)\;=\;\alpha \;+\;\beta_{1}\text{displaced}\;+\;\beta_{2}\text{camp}\;+\;\beta_{3}\text{age}\;+\;\beta_{4}\text{region}\;+\;\beta_{5}\text{governorate}\;+\;\beta_{6}\text{area}\;+\;\beta_{7}\text{education}\;+\;\beta_{8}\text{wealth}\;+\;\beta_{9}\text{parity}\;+\;\beta_{10}\text{media}\;+\;\beta_{11}\left(\text{displaced}\;\;\text{education}\right)$$

## 3 Results

### 3.1 Characteristics of married women aged 15–49 in the 2018 Iraq MICS sample

82% of married women aged 15–49 years in the 2018 Iraq MICS sample lived in Federal Iraq, while 18% lived in the KRI. 70% lived in urban areas, with the remaining 30% in rural areas. With regards to migration, 43% were in households that had continuously lived in the same place. Of those households where the head had moved since birth (*n* = 11,181), 28% reported that they moved for a reason associated with displacement, compared to 72% who moved for other reasons. Of those who had moved, 2% reported their last residence to be a camp. Note this is much lower than estimates of the IDP population living in camps in 2018, since the sample excludes current camp populations. Table 4 presents descriptive characteristics (weighted) by displacement-related variables.

**Table 4:** Descriptive characteristics of married women aged 15–49 years in 2018 Iraq MICS sample (weighted), by displacement variables.

		All married women 15–49 years (*n* = 19,597)
		%
Displacement	**Movement**	
	Never moved	43
	Displaced	16
	Other reason for moving	41
	**Main reason for moving (of those who have moved since birth,***n* = **11, 181)**	
	*Displaced*	*28*
	Conflict or violence	14
	Tribal land disputes	1
	Government evictions	1
	Natural disasters	1
	Return home	12
	*Other reason*	*72*
	Economic reasons	35
	Education	1
	Family reunification	17
	Other	19
	**Previous household residence**	
	City	55
	Town	23
	Rural	20
	Camp	2

### 3.2 Descriptive statistics of modern contraceptive use among married women aged 15–49

**Fig. 1:**
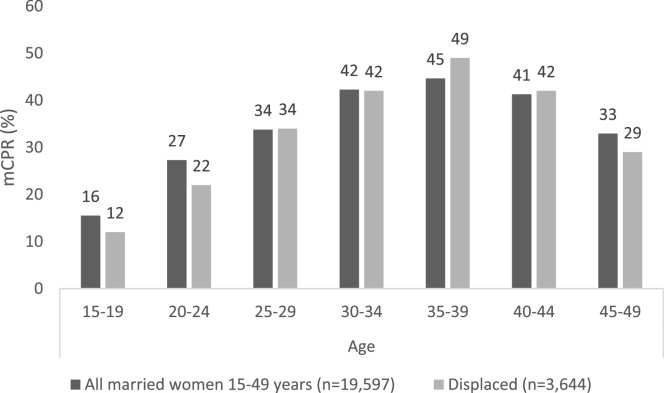
mCPR among all women and displaced women by age group.

36% of married women aged 15–49 currently used at least one modern method of contraception. Modern contraceptive use varied by background characteristics. Modern contraceptive use was lowest among the 15–19 age group (16%) and highest among the 35–39 age group (45%). The outcome also varied by geographic area, being higher in Federal Iraq (38%) compared to the KRI (26%), but relatively similar across rural and urban areas. Women in the poorest quintile reported higher modern contraceptive use (40%) than women in the richest quintile (29%). Across levels of education, modern contraceptive use was highest for women with primary education (38%) and lowest for those with upper secondary education or higher (31%). Modern contraceptive use was highest among women with higher parity. Among households where the head had not continuously lived in the same place since birth, modern contraceptive use was lower among women from displaced households compared to those who moved for other reasons, and among those who previously lived in a camp. The pattern also varied by age group, with a larger difference among those under 25 years old, as illustrated in Fig. 1.

### 3.3 Factors associated with modern contraceptive use

#### 3.3.1 Model 1 – displacement explanatory variables

In the bivariate model, the odds of modern contraception use were 15% lower for displaced women compared to those moving for other reasons (OR = 0.85; 95% CI 0.75–0.97) (see Table 5). When compared to households who had continuously lived in the same place since birth, the odds of modern contraception use were 16% higher among displaced women (OR = 1.16; 95% CI 1.02–1.33), and 36% higher among those moving for other reasons (OR = 1.36; 95% CI 1.23–1.50). The odds of using modern contraception were 34% lower for women who previously lived in a camp compared to other residences (OR = 0.66; 95% CI 0.46–0.97).

**Table 5:** Factors associated with modern contraceptive use among married women aged 15–49 years in Iraq (weighted).

		**All married women 15-49 years**									
		Model 1: bivariate (displacement)				Model 2: multivariate		Model 3: multivariate with interactions			
		*Reason for last move*		*Previous household residence*				*Area^reason for last move*		*Education^reason for last move*	
Factor associated with modern contraceptive use		*Odds ratio*	*95% confidence interval*	*Odds ratio*	*95% confidence interval*	*Odds ratio*	*95% confidence interval*	*Odds ratio*	*95% confidence interval*	*Odds ratio*	*95% confidence interval*
Displacement	**Main reason for moving**										
	Displaced	0.85*	0.75, 0.97			0.90	0.76, 1.07	0.82*	0.68, 0.99	0.63*	0.45, 0.89
	Other reason	Ref.				Ref.		Ref.		Ref.	
	**Previous household residence**										
	Camp			0.66*	0.46, 0.97	0.56*	0.39, 0.80	0.55*	0.38, 0.80	0.55*	0.38. 0.80
	City, town or rural			Ref.		Ref.					
Demographic	**Age, years**										
	15–19					3.17*	1.77, 5.67	3.20*	1.79, 5.71	3.19*	1.80, 5.66
	20–24					3.00*	2.20, 4.08	3.02*	2.22, 4.11	2.97*	2.18, 4.05
	25–29					2.23*	1.76, 2.84	2.25*	1.77, 2.86	2.25*	1.76, 2.87
	30–34					2.33*	1.85, 2.95	2.35*	1.86, 2.97	2.32*	1.83, 2.94
	35–39					2.15*	1.73, 2.67	2.16*	1.74, 2.68	2.15*	1.73, 2.67
	40–44					1.57*	1.23, 1.99	1.58*	1.24, 2.00	1.56*	1.23, 1.99
	45–49					Ref.		Ref.		Ref.	
	**Region**										
	Kurdistan					Ref.		Ref.		Ref.	
	Federal Iraq					1.78*	1.16, 2.72	1.73*	1.14, 2.62	1.81*	1.19, 2.77
	**Area**										
	Urban					Ref.	Ref.		Ref.		
	Rural					0.92	0.77, 1.08	0.81*	0.67, 0.98	0.92	0.78, 1.09
	**Governorate**										
	Baghdad					Ref.		Ref.		Ref.	
	Ninewa					0.76	0.57, 1.01	0.76	0.57, 1.01	0.76	0.57, 1.02
	Sulaimaniyah					1.03	0.49, 2.20	1.05	0.50, 2.21	1.07	0.51, 2.26
	Kirkuk					0.90	0.64, 1.26	0.90	0.64, 1.23	0.90	0.64, 1.26
	Erbil					Omitted because of collinearity					
	Diyala					0.80	0.56, 1.15	0.81	0.56, 1.17	0.79	0.55, 1.14
	Anbar					1.23	0.93. 1.63	1.18	0.90, 1.55	1.20	0.91, 1.58
	Babil					0.82	0.63, 1.07	0.86	0.65, 1.12	0.82	0.62, 1.07
	Duhok					0.90	0.54, 1.49	0.89	0.55, 1.46	0.90	0.54, 1.50
	Karbala					0.95	0.76, 1.20	0.98	0.78, 1.23	0.94	0.75, 1.19
	Wasit					0.94	0.68, 1.31	0.98	0.72, 1.33	0.94	0.67, 1.30
	Salah al-Din					0.81	0.62, 1.04	0.77	0.60, 1.00	0.80	0.62, 1.04
	Najaf					0.75*	0.59, 0.96	0.77*	0.60, 0.99	0.76*	0.59, 0.97
	Qadisiyah					0.76*	0.60, 0.98	0.78	0.61, 1.00	0.76*	0.60, 0.98
	Muthana					1.16	0.66, 2.06	1.18	0.67, 2.06	1.16	0.65, 2.05
	Thi Qar					0.62*	0.46, 0.83	0.63*	0.46, 0.84	0.60*	0.45, 0.82
	Missan					0.86	0.62, 1.18	0.88	0.63, 1.22	0.85	0.62, 1.17
	Basra					0.85	0.68, 1.07	0.87	0.69, 1.10	0.86	0.68, 1.08
	**Parity**										
	0					Ref.		Ref.		Ref.	
	1					53.03*	23.40, 120.21	53.01*	23.40, 120.10	53.71*	23.68, 121.86
	2					119.15*	52.55, 270.15	119.33*	52.63, 270.54	120.58*	53.17, 273.45
	3					192.37*	84.55, 437.70	193.18*	84.89, 439.61	193.63*	85.14, 440.34
	4					295.62*	128.46, 680.27	297.27*	129.15, 684.23	298.52*	129.76, 686.74
	5+					443.35*	193.09, 1017.97	447.02*	194.65, 1026.60	449.46*	195.63, 1032.64
Socioeconomic	**Educational level**										
	Pre-primary or none					Ref.		Ref.		Ref.	
	Primary					1.20*	1.02, 1.42	1.20*	1.02, 1.41	1.10	0.91, 1.34
	Lower secondary					1.13	0.90, 1.41	1.12	0.90, 1.40	0.95	0.74, 1.22
	Upper secondary					1.50*	1.15, 1.95	1.49*	1.14, 1.94	1.35	0.98, 1.85
	**Wealth quintile**										
	Poorest					Ref.	Ref.		Ref.		
	Second					0.92	0.77, 1.10	0.91	0.76, 1.10	0.93	0.77, 1.11
	Middle					0.96	0.78, 1.18	0.97	0.79, 1.19	0.97	0.79, 1.20
	Fourth					0.94	0.75, 1.17	0.95	0.76, 1.19	0.95	0.76, 1.19
	Richest					0.99	0.76, 1.30	1.01	0.77, 1.33	1.00	0.76, 1.32
	**Frequency watching television**										
	Not at all					Ref.		Ref.		Ref.	
	<1 per week					1.49	0.91, 2.44	1.48	0.91, 2.42	1.46	0.90, 2.39
	≥1 per week					1.59*	1.40, 2.23	1.58*	1.13, 2.20	1.56*	1.12, 2.18
	Almost every day					1.50*	1.09, 2.05	1.48*	1.08, 2.03	1.45*	1.06, 1.99
Interactions	Urban^displaced							Ref.			
	Rural^displaced							1.41*	1.07, 1.86		
	Pre-primary or none^displaced									Ref.	
	Primary^displaced									1.39	0.98, 1.97
	Lower secondary^displaced									2.00*	1.34, 2.99
	Upper secondary^displaced									1.50	0.96, 2.33

* Factors that were statistically significant at the 5% level are indicated with an asterisk. Ref. corresponds to the reference category.

#### 3.3.2 Model 2 – displacement, demographic, and socioeconomic explanatory variables

The second logistic regression model included all relevant explanatory variables for modern contraceptive use, including displacement, demographic, and socioeconomic characteristics. Controlling for age, region (Federal Iraq vs KRI), rural/urban area, governorate, education, wealth, parity, exposure to television, and previous residence type, the odds of using modern contraception were 10% lower for displaced women compared to other reasons for moving (OR = 0.90; 95% CI 0.76–1.07), but this was not significant. Factors that were significantly associated with modern contraceptive use in this model were previous residence type (camp), age, region, parity, certain education levels, exposure to television, and three out of 18 governorates.

#### 3.3.3 Model 3 – full model with interactions

**Fig. 2:**
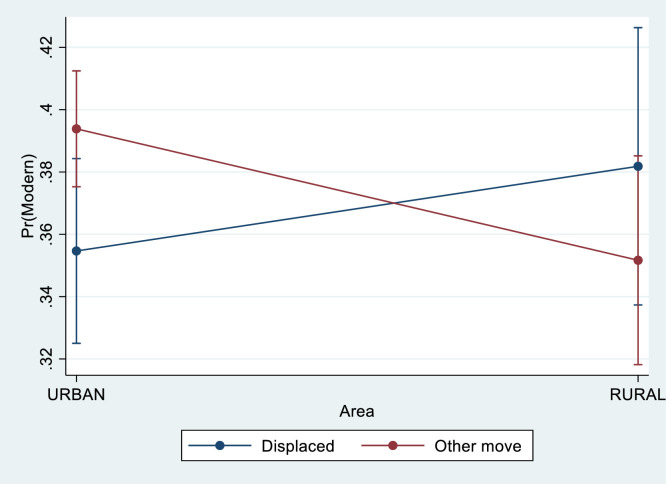
Predictive margins of modern contraceptive use across urban/rural areas, depending on reason for move, controlling for background characteristics (with 95% CIs).

**Fig. 3:**
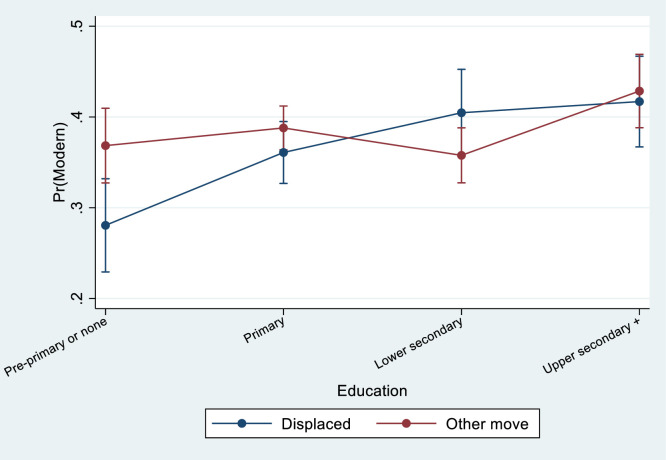
Predictive margins of modern contraceptive use across education levels, depending on reason for move, controlling for background characteristics (with 95% CIs).

The final set of logistic regression models expanded model 2 by including all explanatory variables for modern contraceptive use as well as interactions. I found that the interactions between displacement (reason for moving) and (1) education and (2) urban/rural area were significant at the 5% level. In other words, a woman's level of education and urban/rural area moderated the association between displacement and contraceptive use, controlling for the other demographic and socioeconomic explanatory variables. I also checked for interactions between displacement and other background factors, including previously living in a camp, but found that they did not change the association with modern contraceptive use. Figs. 2 and 3 illustrate these interactions graphically.

## 4 Discussion

### 4.1 Methodological potential and substantive value of household surveys for understanding reproductive outcomes among displacement-affected populations

This paper proves feasibility to use national household survey data to test associations between displacement and modern contraceptive use, controlling for other demographic and socioeconomic characteristics. The three models - bivariate, multivariate, and with interactions - showed a small negative association between displacement and modern contraceptive use, among those who had ever moved. In the bivariate model, the predicted probability of using modern contraception was lower for women from displaced households (36%) compared to those who moved for other reasons (40%). This is only a slight difference but is consistent with emerging evidence of different health outcomes among IDPs compared to other migrants (Cantor et al., 2021). It was surprising and notable to find that the odds of using modern contraception were lowest for households who had never moved; those who cannot move may be the most vulnerable, as moving requires resources (International Committee of the Red Cross, 2000). This highlights the value of comparative analyses using national data and would be worth exploring further. When controlling for other demographic and socioeconomic characteristics (model 2), the main effect of displacement on modern contraceptive use was not significant at the 5% level. However, previously living in a camp was strongly significant, and almost halved the odds of using modern contraception compared to households who previously lived in a city, town or rural area. This finding challenges the notion that populations living in camps have better health outcomes (Whitmill et al., 2016) due to easier access to humanitarian services. Indeed, evidence from Iraqi refugees in Jordan suggests that women feared using camp services in case of negative consequences, such as deportation (Chynoweth, 2008). Missing civil documentation may also be an issue, since women must present a marriage certificate to receive reproductive health services in camps (Tull, 2020). The third set of models were perhaps the most interesting, showing that the association between displacement and modern contraceptive use depends on the women's level of education and area of current residence (urban/rural). This reflects the heterogeneity of IDP populations, and how different vulnerabilities and risks may be experienced in a variety of ways depending on personal situations (Balinska et al., 2019).

Higher odds of using modern contraception were associated with region (Federal Iraq compared to KRI), higher levels of parity, upper secondary and primary education (compared to no education), and regular exposure to television (compared to none). Controlling for other characteristics, women with upper secondary education or higher were almost 50% more likely to use modern contraception compared to those with pre-primary or no education. This aligns with evidence that lack of education opportunities is contributing to high adolescent fertility rates in Iraq (World Bank Group, 2017). However, there was not a significant association between wealth and modern contraceptive use, nor any interaction between wealth and displacement. This challenges existing evidence that contraceptive use is higher among women of higher socioeconomic groups (Agha and Rasheed, 2007; Ismael and Sabir Zangana, 2012), and could imply that poorer women are more likely to receive coercive care (Senderowicz, 2019). It is not possible to account for the role of pharmacies and other private providers of contraception in the MICS data. Private providers may play a key role given the decline of the health system, with variation across socioeconomic groups and the ability to pay for commodities (Tull, 2020). It was surprising that women living in Federal Iraq were more likely to use modern contraception than those in KRI, when controlling for other background characteristics. This is despite the assumption that access to healthcare is better in KRI (Aboulenein and Levinson, 2020). These substantive findings highlight both the importance and feasibility of adopting a displacement lens when analysing reproductive health outcomes in displacement-affected populations.

### 4.2 Limitations of these methods for measuring reproductive health outcomes through a displacement lens

While national household surveys show promising value for comparative analyses of health outcomes among IDPs, these analyses illustrate the challenges and limitations. For the purposes of this discussion, I draw on total survey error, a dominant paradigm in survey research that captures the potential errors of sample survey statistics (Groves and Lyberg, 2010) and feminist approaches to data (D'Ignazio and Klein, 2020).

In terms of *coverage error*, some key groups of interest were excluded from the 2018 MICS survey population. These include people currently living in government or UN-managed displacement camps. At the sampling stage, six conflict-affected districts that were inaccessible to the survey team, but disproportionately affected by displacement, were also excluded (Ba'aj, Al-Hadar, Telafer, Sinjar, Makhmoor, and Haweja). This means the estimates are not necessarily representative of all IDPs in the country and there may be a bias in analysing data at national level (UNHCR, 2020). Furthermore, the question on contraceptive use was only administered to married women aged 15 to 49 years, which may mask vulnerabilities among unmarried women and men. The exclusion of younger adolescents is also important, considering the evidence on early marriage in humanitarian settings (Iraq Ministry of Planning, 2012; Lafta et al., 2018; Baird et al., 2022; Save the Children, 2019). As such, the survey sample likely offers a lower bound set of data which may underestimate the observed association between displacement and modern contraceptive use.

The two displacement measures also have limitations. One of the key challenges is that displacement questions were only administered in the 2018 Iraq MICS household questionnaire, rather than the individual women's questionnaire. While there are some female-headed houses in Iraq, estimated at 11% of total households in 2011 (Iraq Ministry of Planning, 2012), 9% in 2018 (Iraq Central Statistical Organisation, Kurdistan Regional Statistics Office, 2019), and 14% of displaced households living in camps in the KRI in 2017 (Kurdistan Regional Statistics Office, 2018), the majority are male-headed. This means that respondents to the household questionnaire and individual women's questionnaire were likely to be different individuals. As such, household (male) displacement acted as a proxy for women's experiences in most cases - a reflection of how current measurement approaches favour counting some over others. In reality, displacement experiences are not homogenous within a household. Feminist approaches reinforce this norm, arguing that “men's information is too often presented as a group's reality” (Reiter, 1975). Secondly, both displacement measures are based on the last move, rather than previous moves. As such, it is not possible to distinguish between single or multiply displaced households, or those who were displaced but then subsequently moved for other reasons. Thirdly, the ‘camp’ variable only captures those who previously lived in a camp, telling us nothing about the camp population at the time of the survey (who were excluded from the sample). The measures also say nothing about duration of displacement nor place of birth. Finally, the categories mask the complexities of the IDP label. For example, respondents who reported “return home” may include returnees as well as households returning home for reasons other than displacement.

There are also measurement challenges for the response variable, mCPR. Firstly, feminist demographers argue that such dominant population-level outcome indicators say nothing about the quality of the contraceptive services, access, or reproductive rights (including intentions to space or limit pregnancies) (Senderowicz, 2020). Instead, they propose alternative indicators for routine measurement, such as contraceptive autonomy (Senderowicz, 2020). Unfortunately, these types of indicators are not yet measured in household surveys such as MICS. Secondly, data on contraceptive use may have been compromised by interviewer or other contextual effects on reporting, especially for sensitive topics such as contraceptive use (Tourangeau et al., 2000). Existing literature indicates that women in couples use contraception without their partners’ knowledge (Choiriyyah and Becker, 2018), as objection by husbands can be a reason for not using contraception (Ebrahim and Muhammed, 2011). Analysis of the Iraq 2018 MICS metadata possibly supports this; self-reported use of modern contraceptives was lowest in interviews where others were present during the entire interview (31%), compared to when the entire interview was completed in private (37%). Triangulation with additional data, such as that from local health centre registers, could help to check underreporting (Guyavarch, 2006).

There is also the potential for analytic error (West et al., 2017). The analyses are limited by common flaws of regression models such as endogeneity and the inability to draw causal inferences (Agresti, 2018). There could be omitted variable bias due to factors that were not measured in the MICS or not included in the model. These include explanatory variables such as husband's approval of contraceptives, exposure to information about reproductive health, and geographic distance to health services (Stephenson et al., 2007). Other omitted variables relevant to displacement could include possession of civil documentation.

Moving beyond statistical errors, it is useful to consider what we cannot learn from this evidence. Whilst the numbers establish an association between modern contraceptive use and displacement, and identify variation across background characteristics, they omit the lived realities of individuals and communities. There is a need to complement large quantitative datasets with more contextualised and nuanced qualitative evidence on the experiences of IDPs (Chemaly et al., 2016; Cardona-Fox, 2021). This aligns with calls for stronger social analysis of reproductive health, with greater attention to context, as well as the appropriateness, acceptability and uptake of interventions (Price and Hawkins, 2007). For example in Iraq, information on the reproductive health needs and priorities of adolescents and youth is absent (UNICEF, 2018; Jennings et al., 2019). The metrics in this study are a critical source of information, speaking to the measurement needs of global health actors. Yet they are just one form of evidence and could be used as a departure point for designing qualitative research questions.

While Iraq is an extreme case of internal displacement and protracted humanitarian needs driven by conflict, it should not be seen as representative of all displacement contexts. Other drivers of internal displacement, such as rapid-onset emergencies or natural disasters, may intersect with contraceptive use in different ways, and have alternative implications for measurement.

## 5 Conclusions

This study is the first attempt to use national household survey data to analyse modern contraceptive use through the lens of displacement in Iraq. It optimises the available - albeit imperfect - data to offer both substantive and methodological contributions to the literature, as well as implications for policy.

In the early stages of Iraq's new Family Planning and Birth Spacing Strategy, this study highlights inequalities in modern contraception use among married women of reproductive age in Iraq. It draws attention to the reproductive health outcomes of displaced people, highlighting key gaps in knowledge and services that warrant further attention by researchers and policy makers. For example, women from displaced households, especially out-of-camp IDPs who previously lived in camps, may require specific support to meet their reproductive needs.

Methodologically, this study illustrates how household survey data can be used to test associations between important but difficult to measure areas - contraceptive use and displacement - in a humanitarian context. It shows feasibility to construct two displacement-related indicators at the household level, and their incorporation as explanatory variables in logistic regression models. In the current global health environment where metrics are the dominant language, we need to optimise the available data and dig beyond national-level trends. Introducing population displacement brings analytic complexities, but with at least 82 million people forcibly displaced globally (UNHCR, 2021), it can no longer be ignored.

This study also highlights the limitations of existing survey data for measuring reproductive health outcomes among displacement-affected populations. Using the total survey error paradigm offers a structured theoretical framework that could be replicated and offer transferable lessons for other contexts. Feminist approaches to data highlight key problematic areas, such as using household (male) displacement as a proxy for women's experiences, and the narrow focus of measures such as modern contraceptive use. National household surveys could be an important source of evidence on IDP health outcomes, but there is still some way to go to ensure no one is left behind (UN General Assembly 2015).

## Funding

This work was supported by the Economic and Social Research Council as part of a 1+3 doctoral grant (ES/P000622/1).

## Data access statement

Multiple Indicator Cluster Survey (MICS) datasets are openly available from UNICEF pending free registration at https://mics.unicef.org/surveys.

## CRediT authorship contribution statement

**Rosanna Le Voir:** Visualization, Writing – original draft.

## Declaration of Competing Interest

The author declares that they have no competing interests.
